# Minimum Dietary Diversity for Women: Partitioning Misclassifications by Proxy Data Collection Methods using Weighed Food Records as the Reference in Ethiopia

**DOI:** 10.1016/j.cdnut.2024.103792

**Published:** 2024-06-13

**Authors:** Giles T Hanley-Cook, Sara Hoogerwerf, Juan Pablo Parraguez, Simone M Gie, Bridget A Holmes

**Affiliations:** Food and Nutrition Division (ESN), Food and Agriculture Organization of the United Nations (FAO), Rome, Italy

**Keywords:** noninferiority, measurement agreement, food groups, list-based recall, 24-h recall, recall bias

## Abstract

**Background:**

Nonquantitative list-based or open 24-h recalls (24-HRs) have been shown to overestimate the prevalence of Minimum Dietary Diversity for Women (MDD-W), as compared with direct quantitative observations. However, the main sources of error are unknown.

**Objectives:**

To assess the measurement agreement of proxy data collection methods for MDD-W, as compared with weighed food records (WFRs).

**Methods:**

Applying a noninferiority design, data were collected from 431 nonpregnant females in Ethiopia. MDD-W estimates from both proxy data collection methods were compared with the WFR prevalence by McNemar’s chi-square tests, Cohen’s Kappa, and receiver operator characteristic analyses. Ten-point food group diversity scores (FGDS) were compared by Bland–Altman plots, Wilcoxon matched-pairs tests, and weighted Kappa. Food group misclassifications were partitioned into errors related to respondent biases or the questionnaire development.

**Results:**

List-based and open 24-HRs overreported MDD-W by 8 and 4 percentage points, respectively, as compared with WFR (objective MDD-W prevalence: 8%). Furthermore, list-based 24-HRs overestimated FGDS by 0.4 points (limits of agreement [LOA]: −1.1, 2.0), whereas open 24-HRs led to a 0.3 point (LOA: −1.2, 1.7) overestimate. Food groups most likely to be misreported using proxy data collection methods were “pulses,” “nuts and seeds,” “dairy products,” and “other fruits.” Underreporting of consumption occurred among <4% of females for all food groups. Furthermore, respondent biases were the predominant cause of food group overreporting, except for the “pulses” and “other vegetables” food groups, where food items incorrectly included on the food list were the main source of errors.

**Conclusions:**

Food group consumption misclassifications by proxy data collection methods were mainly attributable to females overreporting consumption because of respondent biases or the criterion for foods to be counted, rather than the suboptimal development of the food list in Ethiopia. To obtain precise and accurate MDD-W estimates at the (sub)national level, rigorous context-specific food list development, questionnaire pilot testing, and enumerator training are recommended to mitigate identified biases.

## Introduction

Quantitative or semiquantitative assessment methods commonly used to estimate individual-level dietary intake, such as food frequency questionnaires, food diaries, and (repeated) 24-h recalls (24-HRs), require highly proficient enumerators, resource-intensive data collection, processing, and analysis, and the availability of comprehensive food composition tables or databases [1]. To facilitate timely monitoring and evaluation of nutrition-sensitive programs, policies, and diets at national-level and cross-sectoral communication, there is a demand for non-quantitative proxy indicators that capture ≥1 core component of healthy diets (for example, nutrient adequacy, macronutrient balance, diversity, and moderation) [2]. To ensure the uptake and scalability of such proxy dietary indicators across contexts and time, indicators must be accurate, precise, affordable, and straightforward to interpret for decision-makers [3].

To help fulfill these extensive requirements, the dichotomous food group-based Minimum Dietary Diversity for Women (MDD-W) indicator, which is the intake of foods from ≥5 distinct food groups, was developed for use in low- and middle-income countries (LMICs) [4]. In resource-poor settings, such as rural Ethiopia, the usual diets of nutritionally vulnerable population groups are monotonous, nondiverse (that is, predominantly based on starchy staples), unbalanced, and subsequently provide insufficient levels of bioavailable micronutrients [5]. Hence, MDD-W was initially validated to signal a minimally acceptable level of micronutrient adequacy among non-pregnant females and has been widely used as a population-level indicator of dietary diversity [6]. Following the FAO’s updated guidance, MDD-W should be enumerated by either nonquantitative list-based or open 24-HR, which are hereafter referred to as “proxy data collection methods” [4].

Nonetheless, Hanley-Cook *et al.* [7] indicated that both proxy data collection methods—but in particular the list-based 24-HR—are prone to substantial overreporting of food group consumption, as compared with objectively measured intakes in 3 LMICs. Moreover, previous studies using direct observation or known weights of foods consumed support that misclassifications from (quantitative) dietary recall methods differ according to specific food types (for example, snacks and beverages), physical forms (for example, single unit and amorphous), and food groups (for example, vegetables and fruits) [[8], [9], [10]]. Without an objective measure, such as a recovery biomarker or a direct measurement of individual-level intake, systematic respondent biases cannot be identified, which hampers mitigation actions including the methodological refinement of survey tools [9].

Therefore, to comprehend the sources of food group misreporting, this study leveraged data from a noninferiority study comparing the proxy data collection methods against weighed food records (WFRs) in rural Ethiopia. To this end, we assessed food group misclassification errors by identifying food or beverage item contributors and quantified the magnitude and variability of the errors both within and between the food groups underlying MDD-W [11]. In addition, this study examined the misclassification errors specifically related to the 15 g/d minimum quantity threshold for a food group to “count” toward MDD-W. In other words, this study aimed to identify if the MDD-W questionnaire incorrectly included food item examples that were frequently consumed in trivial quantities [4].

This research thus aims to partition the multiple sources of food group under and overreporting—and consequently biased MDD-W estimates—in the Amhara Region of Ethiopia, with the overarching objective to inform generalizable strategies that could improve the accuracy of MDD-W data collection in LMICs (for example, food list development, enumerator training, and pilot-testing).

## Methods

Our research is reported using the STROBE-nut checklist [12].

### Study design

The complete multicountry study—including sample size computation, study populations, data sources, preparatory phase (including pre and pilot testing), and data collection methods—used in the current study has been described in detail elsewhere [7].

The objective of the current research was to determine whether proxy data collection methods are comparable with a WFR for capturing the proportion of nonpregnant females achieving MDD-W. For this purpose, in the rainy season between June and August 2019, WFR data (day 1) were collected from 431 nonpregnant females aged 15–49 y in the Amhara Region of Ethiopia in 24 villages (*gotes*) in 13 subdistricts (*kebeles*) from 3 districts (*woredas*; that is, Debre Berhane, Shao Robit, and Tehuledere), characterized by rural, periurban, and urban residencies. Subsequently, the proxy data collection methods were both enumerated on the day after the WFR (day 2), but by different interviewers and at different times (both at random, the latter corresponding to either the morning or afternoon). Investigators collecting, processing, and analyzing the nonquantitative self-reported dietary intake data were not present during the collection of observed dietary intake data.

Before data collection, the food list was adapted to the local context with the key aim of excluding sentinel foods frequently consumed in trivial quantities. To this end, focus group discussions with nutritionists from the Ethiopian Public Health Institute and local health workers were conducted, and data from a previous study that collected MDD-W using nonquantitative open 24-HRs in Ethiopia were reanalyzed [13]. The initial list-based questionnaire was pilot-tested among 50 females. Minor amendments were made to the food list following the pilot test, such as the inclusion of *chechebsa* as a sentinel food for the “foods made from grains” food subgroup and the exclusion of lemon as a sentinel food from the “other fruits” food group. The final list-based 24-HR module (and food list for open 24-HR) used in the present study was subsequently published in Appendix 5 (pages 152–153) of the 2021 *Minimum Dietary Diversity for Women: An Updated Guide for Measurement* [4].

The number of households sampled from each *woreda* was determined using the probability proportional to size (PPS) method (that is, 97–164 nonpregnant females from each *woreda*). Moreover, a 3-staged stratified systematic random sampling approach was used for the selection of *kebeles* (that is, 3–5 from each *woreda*, also using PPS), *gotes* (that is, 1–2 from each *kebele*), and households (that is, 5–22 from each *gote*).

### Food group diversity score and the MDD-W indicator

Where applicable, food items were assigned to 1 of 17 required food subgroups enumerated through MDD-W survey modules. The 17 food subgroups were subsequently aggregated into the 10 predefined MDD-W food groups [4]: *1*) starchy staple foods; *2*) beans, peas, and lentils (henceforth referred to as “pulses”); *3*) nuts and seeds; *4*) dairy products (milk, yogurt, and cheese); *5*) flesh foods (meat, fish and seafood, poultry, and liver or organ meats); *6*) eggs; *7*) dark green leafy vegetables; *8*) vitamin A-rich fruits and vegetables; *9*) other vegetables; and *10*) other fruits. For the WFR, the food group diversity scores (FGDS) (0–10 points) were constructed by summing the number of food groups consumed in quantities of ≥15 g/d. For the proxy data collection methods, the FGDS was calculated by summing the number of food groups from which food items on the food list were recalled as being consumed. During the context-specific adaption phase, food items usually consumed in trivial quantities (that is, <15 g/d) were excluded from the food list. Achieving MDD-W was defined as a nonpregnant female consuming ≥5 food groups. Other food items were assigned to 13 additional food groups for the WFR and nonquantitative open 24-HR also enumerated through MDD-W survey modules [4]: *1*) packaged salty snacks, *2*) deep fried snacks, *3*) instant noodles, *4*) fast food restaurant foods, *5*) sweet foods, *6*) sugar-sweetened beverages, *7*) sweetened infusions; *8*) insects and small protein foods, *9*) wild plants, *10*) red palm oil, *11*) other oils and fats, *12*) condiments and seasonings, and *13*) other beverages and foods. The collection of food groups *1*)–*7*) is recommended, whereas *8*)–*13*) are optional [4].

### Statistical analysis

Data management and statistical analysis were conducted in Stata version 16.1 [14]. Descriptive data are presented as means ± SDs and median (*P*^25^, *P*^75^) or frequency (percentages).

To assess the effects of the allocation sequence of proxy data collection methods on MDD-W and FGDS (that is, potential “carry over” effect [15,16]), adjusted logistic and linear regression models were fitted, respectively. To clarify, models specified the list-based 24-HR estimates of either MDD-W or FGDS as the dependent variable and included an interaction term between the related open 24-HR estimate and binary allocation sequence (that is, open 24-HR first and list-based 24-HR second on day 2, or vice versa) [17].

In parallel to Hanley-Cook et al. [7], we used McNemar’s chi-square tests for paired proportions to evaluate how well both proxy data collection methods estimated MDD-W and individual food group consumption compared with WFR. To measure agreement among females achieving MDD-W, we used simple Cohen’s Kappa statistics. Kappa scores (*κ*) of 0.21–0.40 indicate fair agreement, 0.41–0.60, moderate agreement, 0.61–0.80, substantial agreement, and 0.81–1.00, almost perfect agreement [18]. We also performed a “gold standard” receiver operator characteristic analysis to compare how well list-based and open 24-HRs predicted MDD-W as compared with WFR. The AUC summarizes the predictive power of MDD-W for the proxy data collection methods. An AUC significantly different from 0.5 and ≥0.70 was deemed satisfactory to indicate accuracy [19].

In addition, we used Bland–Altman plots to assess the measurement agreement between FGDS estimated by the 2 proxy data collection methods as compared with the WFR [20]. Limits of agreement (LOA) were calculated as the mean difference ± 1.96 SDs and interpreted as the range where 95% of differences were expected to occur. Furthermore, we used Wilcoxon matched-pairs signed-rank tests and weighted Cohen’s Kappa (*κ*_*w*_) to assess how well both proxy data collection methods estimated FGDS compared with WFR. We quantified the frequency of misreporting (that is, type I and II errors) for MDD-W and each individual food group using 2 × 2 contingency tables and identified the food groups that were most often misreported by the proxy data collection methods.

Lastly, we examined the sources of errors by individual food items, which included *1*) the number of omissions (that is, foods consumed according to the WFR, but not reported) and intrusions (that is, foods reported, but not consumed according to the WFR) for both list-based and open 24-HRs, and *2*) 15 g/d minimum threshold errors, which evaluated the quantities consumed of individual food items. The guiding principle behind MDD-W is to err on the side of not counting food items that are potentially consumed in trivial quantities, to avoid inflating the FGDS, and subsequently MDD-W prevalence [4]. Hence, to determine *post hoc* whether specific food items should have been included or excluded on the predefined Ethiopian food list, we set the arbitrary but stringent criteria that, among consumers of the food item in question, ≥70% had intakes ≥15 g/d.

Statistical significance was set at *P* < 0.05 for all tests, except for interactions tests (*P* < 0.10).

### Ethical approval

The study was approved by the Ethiopian Public Health Institute Scientific and Ethical Review Office (EPHI/IRB/156/2018). Written informed consent was obtained from all study participants.

### Results

The interaction term for the allocation sequence of proxy data collection methods was nonsignificant for the dichotomous MDD-W indicator (*P* = 0.65), whereas statistically significant for FGDS (*P* = 0.029). Nonetheless, the effect modification of enumerating the list-based 24-HR first (*n* = 233, that is, morning of day 1) led only to a 0.15-point higher FGDS for open 24-HR (that is, afternoon of day 2). Therefore, our analyses include all dietary data collected from proxy data collection methods (both *n* = 431).

### Sample characteristics

On average, nonpregnant females were aged 30 ± 7.3 y and 25.9% were lactating. Overall, ∼60% of females were Orthodox Christian and ∼40% were Muslims. Furthermore, males were the head of >80% of households (median household size (*P*^25^, *P*^75^): 4 (3–5) members) and ∼70% of interviewed women were their wives. Moreover, ∼50% of females were homemakers and one-quarter had not received any formal education, whereas ∼35% and ∼25% of females completed primary or secondary education, respectively. None of the sampled females were fasting or subjectively described atypical consumption patterns on the day of the WFR.

### Predictive performance of proxy data collection methods for MDD-W

**FIGURE 1:**
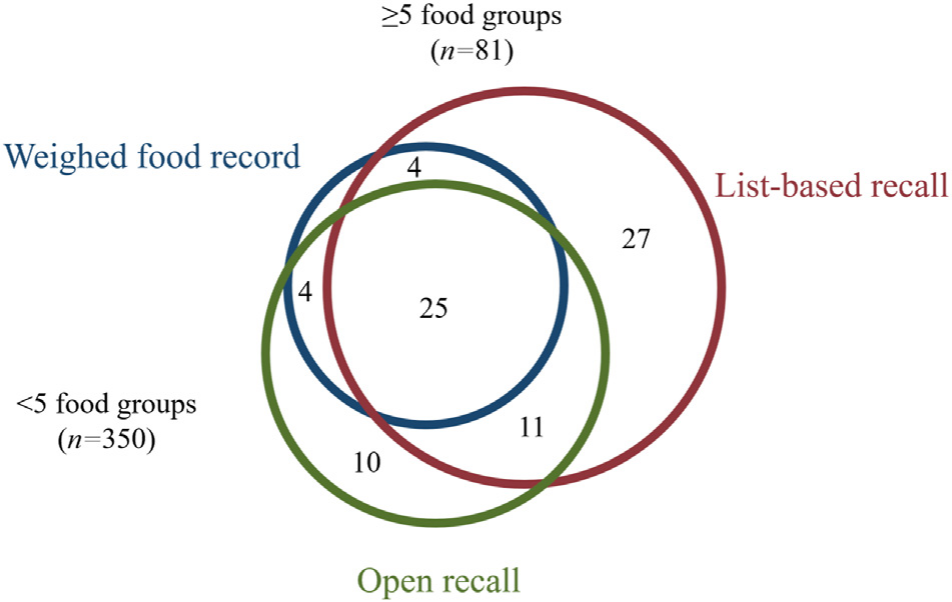
Proportional Venn diagram of nonpregnant females achieving Minimum Dietary Diversity for Women (MDD-W) measured by list-based or open 24-h recalls, as compared with weighed food records in Ethiopia. In total, 350 females did not achieve MDD-W according to all 3 data collection methods, whereas 81 females achieved MDD-W according to ≥1 of the data collection methods.

**FIGURE 2:**
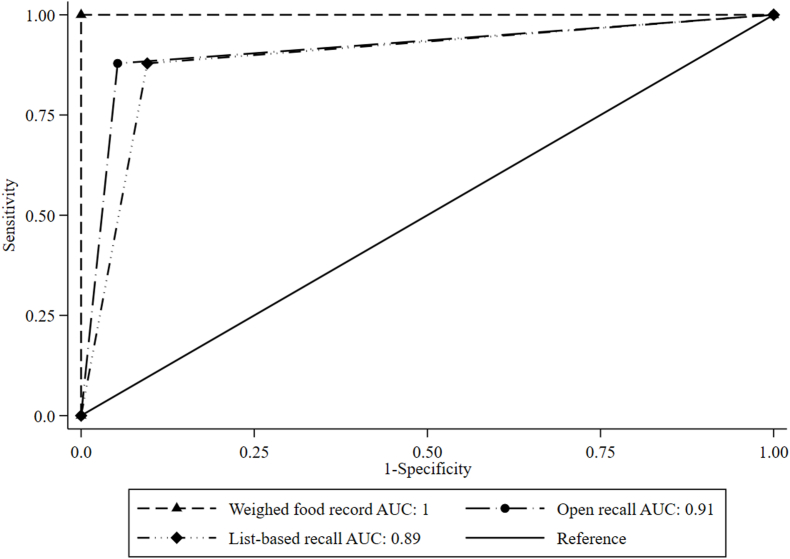
Receiver operating characteristic analysis for dichotomous Minimum Dietary Diversity for Women (MDD-W) measured by list-based and open 24-h recalls, as compared with weighed food records in Ethiopia..

The proportion [95% confidence interval (CI)] of nonpregnant females achieving MDD-W (that is, ≥5 food groups) was 8% (95% CI: 5, 11) as observed by WFRs, whereas according to the list-based 24-HR the prevalence was 16% (95% CI: 12, 19), and 12% (95% CI: 9, 15) using the open 24-HR (both *P* < 0.001; Table 1, Figure 1). The list-based and open 24-HRs correctly classified (that is, true positive and negative values) 90% and 94% of females as (not) achieving MDD-W, respectively (Table 2). Simple Kappa values for the dichotomous indicator signaled moderate agreement for list-based (*κ* = 0.53) and substantial agreement for open 24-HR against WFR (*κ* = 0.67). Furthermore, list-based 24-HR had a sensitivity (that is, truly achieving MDD-W) of 88% and a specificity (that is, truly not achieving MDD-W) of 90%, whereas open 24-HR had a sensitivity and specificity of 88% and 94%, respectively (Figure 2). Moreover, both proxy data collection methods showed statistically comparable predictive capacity for MDD-W (95% CIs AUC: 0.86, 0.97 compared with 0.83, 0.95).

**TABLE 1:** Proportions of nonpregnant Ethiopian females (15–49 y) having consumed food groups and achieved Minimum Dietary Diversity for Women (MDD-W), based on weighed food record, list-based, and open 24-h recalls (*n* = 431)^1^

	Weighed food record	List-based recall	Open recall
All starchy staple foods	100	100	100
Beans, peas, and lentils	80.3	90.3∗∗∗	89.8∗∗∗
Nuts and seeds	0.7	4.6∗∗∗	3.7∗∗∗
Dairy	4.2	7.2∗∗∗	7.2∗∗∗
Flesh foods	8.8	10.7∗	10.7∗
Egg	5.8	6.5	6.0
Dark green leafy vegetables	6.7	11.8∗∗∗	8.7∗
Vitamin-A-rich fruits and vegetables	11.6	15.1∗	11.1
Other vegetables	94.0	96.5	97.2∗
Other fruits	4.4	10.9∗∗∗	6.5
MDD-W	7.7	15.6∗∗∗	11.6∗∗∗

1 McNemar’s chi-square tests for paired proportions were used to assess statistically significant differences between weighed food records and proxy data collection methods. ∗*P* < 0.05, ∗∗*P* < 0.01, and ∗∗∗*P* < 0.001.

**TABLE 2:** Agreement between dichotomous Minimum Dietary Diversity for Women (MDD-W) measured by list-based or open 24-h recalls, as compared with weighed food record in Ethiopia (*n* = 431)

	Weighed food record		Agreement statistics	
	<5 food groups *n* (%)	≥5 food groups *n* (%)	% agreement	Cohen’s kappa
**List-based recall**				
<5 food groups	360 (83.5)	4 (0.928)^2^	90.3	0.53∗∗∗
≥5 food groups	38 (8.82)^1^	29 (6.73)		
**Open recall**				
<5 food groups	377 (87.5)	4 (0.928)^2^	94.2	0.67∗∗∗
≥5 food groups	21 (4.87)^1^	29 (6.73)		

1 False positive finding (type I error). ∗*P* < 0.05, ∗∗*P* < 0.01, ∗∗∗*P* < 0.001.

2 False negative finding (type II error).

### Measurement agreement of proxy data collection methods for FGDS

**FIGURE 3:**
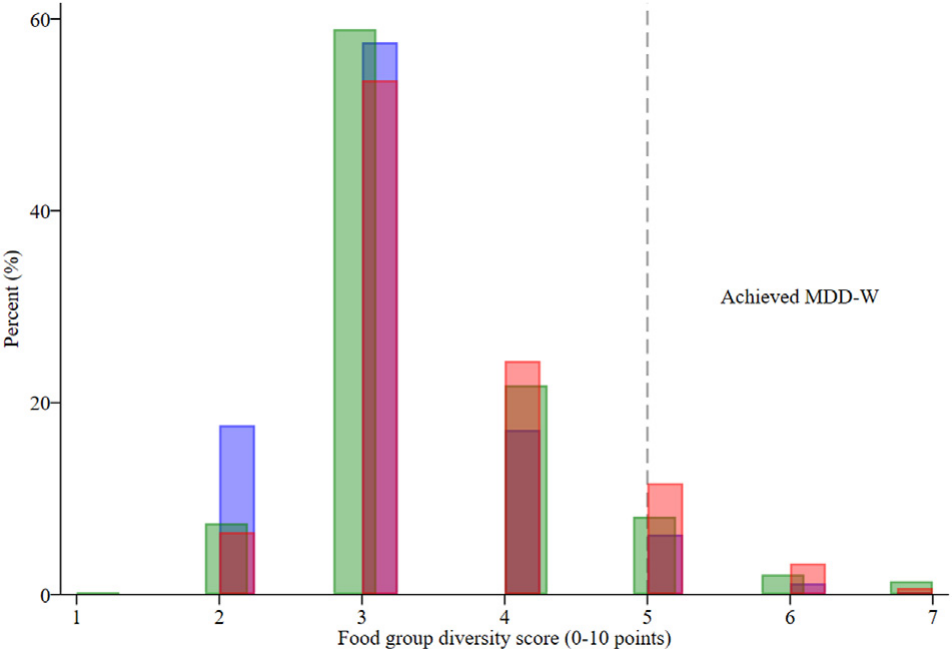
Histogram of food group diversity score (0–10 points) measured by list-based (red) 24-h recall, open 24-h recall (green), and weighed food record (blue) in Ethiopia. MDD-W, Minimum Dietary Diversity for Women.

**FIGURE 4:**
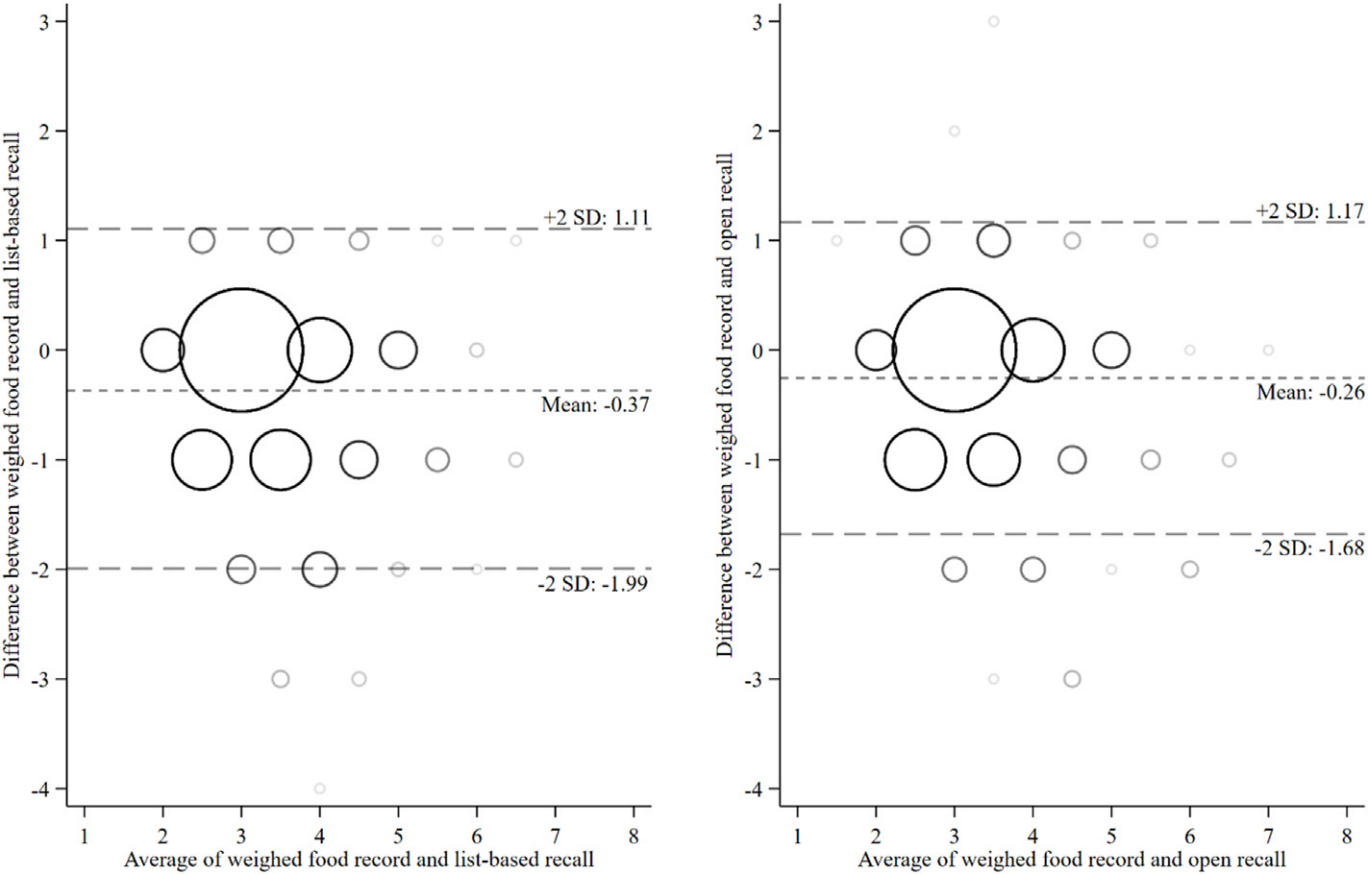
Bland–Altman plot for food group diversity score (0–10 points) measured by list-based or open 24-h recalls, as compared with weighed food record in Ethiopia. The size of the bubbles is proportional to the number of nonpregnant females.

The distributions of FGDS, by dietary assessment method, are presented in Figure 3. In Ethiopia, the median (*P*^25^, *P*^75^) number of food groups was 4 [3,5] by WFR. On average, FGDS was 0.4 points higher for the list-based (LOA: −1.1, 2.0) and 0.3 points higher for open 24-HRs (LOA: −1.2, 1.7) than those of WFR (both *P* < 0.001; Table 3, Figure 4). The percent agreement of FGDS was 91% for list-based and 93% for open 24-HR. The weighted Kappa for FGDS was very similar for list-based and open 24-HRs (*κ*_w_ = 0.50 compared with 0.53, respectively) (Table 3).

**TABLE 3:** Agreement between ordinal food group diversity score measured by list-based or open 24-h recalls, as compared with weighed food record in Ethiopia (*n* = 431)

	Median (*P*^25^, *P*^75^)^1^	% agreement	Weighted Kappa
**Weighed food record (reference)**	3 (3, 3)	—	—
**List-based recall**	3∗∗∗ (3, 4)	90.7	0.50∗∗∗
**Open recall**	3∗∗∗ (3, 4)	93.1	0.53∗∗∗

1 Wilcoxon matched-pairs signed-rank test. ∗*P* < 0.05, ∗∗*P* < 0.01, and ∗∗∗*P* < 0.001.

### Partitioning of food group misreporting by proxy data collection methods

As detailed in Hanley-Cook et al. [7], in Ethiopia the list-based 24-HR reported higher or identical proportions of consumption for most food groups as compared with the WFR and the open 24-HR, except “other vegetables” for the latter (Table 1). Furthermore, for the list-based 24-HR, 83% of the misclassifications for MDD-W were type I (that is, false positive values), as compared with 74% for open 24-HR (Table 2).

The 2 × 2 contingency tables for each of the 10 food groups used to construct MDD-W indicated that for the list-based 24-HR, 69% of type I errors arose from overreporting of “pulses” (26%), “dark green leafy vegetables” (12%), “vitamin A-rich fruits and vegetables” (15%), and “other fruits” (17%) food groups, whereas 35% of type II errors (that is, false negative values) were from underreporting “vitamin A-rich fruits and vegetables” (Table 4). Likewise for open 24-HRs, 66% of type I errors came from overreporting of “pulses” (30%), “dark green leafy vegetables” (12%), “other vegetables” (15%), and “other fruits” (10%) food groups, whereas 31% of type II errors were from underreporting “vitamin A-rich fruits and vegetables” (Table 4).

**TABLE 4:** Agreement between 10 Minimum Dietary Diversity for Women (MDD-W) food groups measured by list-based or open 24-h recalls, as compared with weighed food record in Ethiopia (*n* = 431)

	Weighed food record		Agreement statistics	
	0–14 g/d *n* (%)	≥15 g/d *n* (%)	% agreement	Cohen’s Kappa
	All starchy staple foods			
List-based recall				
<15 g/d	0 (0)	0 (0.0)^2^	100	—
≥15 g/d	0 (0)^1^	431 (100)		
Open recall				
<15 g/d	0 (0)	0 (0)^2^	100	—
≥15 g/d	0 (0)^1^	431 (100)		

	Beans, peas, and lentils			
List-based recall				
<15 g/d	33 (7.66)	9 (2.09)^2^^,^^3^	85.8	0.45∗∗∗
≥15 g/d	52 (12.1)^1^	337 (78.2)		
Open recall				
<15 g/d	37 (8.58)	7 (1.62)^2^^,^^4^	87.2	0.51∗∗∗
≥15 g/d	48 (11.1)^1^	339 (78.7)		

	Nuts and seeds			
List-based recall				
<15 g/d	411 (95.4)	0 (0.)^2^	96.1	0.25∗∗∗
≥15 g/d	17 (3.94)^1^	3 (0.696)		
Open recall				
<15 g/d	415 (96.3)	0 (0.0)^2^	97.0	0.31∗∗∗
≥15 g/d	13 (3.02)^1^	3 (0.696)		

	Dairy			
List-based recall				
<15 g/d	400 (92.8)	0 (0.0)^2^	97.0	0.72∗∗∗
≥15 g/d	13 (3.02)^1^	18 (4.18)		
Open recall				
<15 g/d	400 (92.8)	0 (0.0)^2^	97.0	0.72∗∗∗
≥15 g/d	13 (3.02)^1^	18 (4.18)		

	Flesh foods			
List-based recall				
<15 g/d	384 (89.1)	1 (0.232)^2^	97.7	0.87∗∗∗
≥15 g/d	9 (2.09)^1^	37 (8.58)		
Open recall				
<15 g/d	382 (88.6)	3 (0.696)^2^	96.8	0.82∗∗∗
≥15 g/d	11 (2.55)^1^	35 (8.12)		

	Egg			
List-based recall				
<15 g/d	402 (93.3)	1 (0.232)^2^	98.8	0.90∗∗∗
≥15 g/d	4 (0.928)^1^	24 (5.57)		
Open recall				
<15 g/d	402 (93.3)	3 (0.696)^2^	98.4	0.85∗∗∗
≥15 g/d	4 (0.928)^1^	22 (5.10)		

	Dark green leafy vegetables			
List-based recall				
<15 g/d	377 (87.5)	3 (0.696)^2^	93.5	0.62∗∗∗
≥15 g/d	25 (5.80)^1^	26 (6.03)		
Open recall				
<15 g/d	383 (88.9)	6 (1.39)^2^	94.2	0.62∗∗∗
≥15 g/d	19 (4.41)^1^	23 (5.34)		

	Vitamin-A-rich fruits and vegetables			
List-based recall				
<15 g/d	351 (81.4)	15 (3.48)^2^^,^^5^	89.6	0.55∗∗∗
≥15 g/d	30 (6.96)^1^	35 (8.12)		
Open recall				
<15 g/d	367 (85.2)	16 (3.71)^2^^,^^6^	93.0	0.65∗∗∗
≥15 g/d	14 (3.25)^1^	34 (7.89)		

	Other vegetables			
List-based recall				
<15 g/d	7 (1.62)	8 (1.86)^2^^,^^7^	93.7	0.31∗∗∗
≥15 g/d	19 (4.4)^1^	397 (92.1)		
Open recall				
<15 g/d	2 (0.464)	10 (2.32)^2^^,^^8^	92.1	0.07
≥15 g/d	24 (5.57)^1^	395 (91.6)		

	Other fruits			
List-based recall				
<15 g/d	378 (87.7)	6 (1.39)^2^^,^^9^	90.7	0.35∗∗∗
≥15 g/d	34 (7.89)^1^	13 (3.02)		
Open recall				
<15 g/d	396 (91.9)	7 (1.62)^2^^,^^10^	94.7	0.48∗∗∗
≥15 g/d	16 (3.71)^1^	12 (2.78)		

1 False positive finding (type I error). ∗*P* < 0.05, ∗∗*P* < 0.01, ∗∗∗*P* < 0.001.

2 False negative finding (type II error).

3 2× *shiro* (pea, bean), 3× mature beans, 2× peas, and 2× lentils.

4 2× *shiro* (pea, bean), 2× mature beans, 1× peas, and 2× lentils.

5 5× carrot and 2× ripe mango. Moreover, Ethiopian lettuce, spinach, and Swiss chard were misclassified 2×, 3×, and 3×, respectively (that is, dark green leafy vegetables).

6 7× carrot and 1× ripe mango. Moreover, Ethiopian lettuce, spinach, and Swiss chard were misclassified 2×, 3×, and 3×, respectively (that is, not as dark green leafy vegetables).

7 7× onion and 1× onion and tomato.

8 8×onion and 2× onion and tomato.

9 3× banana, 2× guava, and 1× lemon (not included on the food list).

10 4× banana, 1× banana and orange, 1× guava, and 1× lemon (not included on the food list).

For most food groups, the predominant sources of overreporting (that is, >80% of cases) by both proxy data collection methods were respondent biases. In other words, females reported having consumed the food group while the WFR objectively indicated no food items belonging to the respective food group were consumed (Table 5). In contrast, for the “pulses” [for example, chickpea powder (*shiro*)] and “other vegetables” food groups (for example, onion) the main source of overreporting (that is, >75% of cases) was trivial intake quantities of food items belonging to the respective food group (for example, 1–14 g/d consumed, thus not counted toward food group consumption when using WFR) (Table 5). Furthermore, *shiro*, mature beans, and green peppers were incorrectly included on the extensive context-specific food list as they were consumed in nontrivial quantities (for example, ≥15 g/d) by <60% of consumers (Supplemental Table 1).

**TABLE 5:** Source of overreporting of Minimum Dietary Diversity for Women (MDD-W) food groups measured by list-based or open 24-h recall, as compared with weighed food record in Ethiopia (*n* = 431)

	Weighed food record	
	0 g *n* (%)	1–14 g *n* (%)
	All starchy staple foods	
List-based recall	—	—
Open recall	—	—

	Beans, peas, and lentils	
List-based recall	11 (21.2)	41 (78.8)^1^
Open recall	9 (18.8)	39 (81.2)^2^

	Nuts and seeds	
List-based recall	16 (94.1)	1 (5.88)
Open recall	13 (100)	—

	Dairy	
List-based recall	13 (100)	—
Open recall	13 (100)	—

	Flesh foods	
List-based recall	8 (88.9)	1 (1.11)
Open recall	10 (90.9)	1 (9.09)

	Egg	
List-based recall	4 (100)	—
Open recall	4 (100)	—

	Dark green leafy vegetables	
List-based recall	25 (100)	—
Open recall	19 (100)	—

	Vitamin-A-rich fruits and vegetables	
List-based recall	25 (83.3)	5 (16.7)
Open recall	12 (85.7)	2 (14.3)

	Other vegetables	
List-based recall	—	19 (100)^3^
Open recall	3 (12.5)	21 (87.5)^4^

	Other fruits	
List-based recall	29 (85.3)	5 (14.7)
Open recall	12 (75.0)	4 (25.0)

1 4× *shiro* powder, 19× *shiro* (pea, bean), 12× mature beans, 2× peas, 2× lentils and *shiro* (pea, bean), 1× lentils, and 1× lentils and mature beans.

2 4× *shiro* powder, 18× *shiro* (pea, bean), 12× mature beans, 2× peas, 1× lentils, 1× lentils and mature beans, and 1× lentils and *shiro* (pea, bean).

3 18× onion and 1× onion and tomato.

4 18× onion, 2× onion and green pepper, and 1× onion and tomato.

## Discussion

In Ethiopia, list-based and open 24-HRs overreported FGDS and females achieving MDD-W by ∼8 and 4 percentage points, respectively, as compared with WFR. Food groups most likely to be misreported (that is, predominantly intrusions) using proxy data collection methods were “pulses,” “nuts and seeds,” “dairy products,” and “other fruits.” Furthermore, except for the “pulses” and “other vegetables” food groups, respondent biases, which include memory lapses, recalling intake beyond the 24-h period, and social desirability and approval, were the principal causes of food group overreporting. For the “pulses” food group, *shiro* (powder) and mature beans were food items incorrectly included on the food list, as <70% of consumers had intakes ≥15 g/d. Likewise, for the “other vegetables” food group, green peppers should have been excluded from the food list. Although onion was a food item correctly included on the food list, it was consumed in quantities <15 g/d by ∼10% of consumers. Underreporting of food group consumption was infrequent in the current study.

To our knowledge, this is the only noninferiority study with an objective reference value for MDD-W. Although this is a key strength of the current study, it also makes the main findings challenging to compare to previous research. To clarify, nonquantitative list-based and open 24-HRs and quantitative 24-HRs are subject to correlated errors due to the known carry-over of recall biases [21]. Therefore, when data from quantitative 24-HR are used as the reference value for MDD-W, this likely leads to an underestimate of the total number of misclassifications by proxy data collection methods [11].

Our results are ostensibly in contrast to a multicountry study among 600 pregnant Bangladeshi and 655 pregnant Indian females [19]. To clarify, Nguyen et al. [19] reported that list-based 24-HRs underreported FGDS and MDD-W prevalence as compared with quantitative 24-HRs with >0 or ≥15 g/d minimum intake thresholds for food groups to count. Moreover, in Bangladesh and India, the “pulses” (that is, soybeans) and “other vegetables” (that is, tomato and onion) food groups, respectively, were most susceptible to underreporting when assessing the relative validity of list-based 24-HRs as compared with quantitative 24-HRs [19]. Nevertheless, similar to our results, “flesh foods” and “vitamin A-rich fruit and vegetables” were overreported in Bangladesh, whereas in India list-based 24-HRs overreported the consumption of “nuts and seeds,” “flesh foods,” “dark green leafy vegetables,” and “vitamin A-rich fruits and vegetables” food groups [19].

Nevertheless, similar to our results, Uyar et al. [22] reported that the prevalence of achieving MDD-W was significantly overestimated by 6 percentage points among 488 females in Ethiopia when comparing the relative validity of the Diet Quality Questionnaire (DQQ) to quantitative 24-HRs [22]. Furthermore, likewise to the direction of our findings but in slightly smaller magnitudes, “dairy products,” “vitamin A-rich fruits and vegetables,” and “other fruits” food groups were particularly prone to overreporting by females in Ethiopia [22]. As previously described, the smaller proportion of misclassification errors might be explained by the fact that both the DQQ and quantitative 24-HRs are subject to similar respondent biases, which would be additionally captured as intrusions or exclusions when comparing proxy data collection methods for MDD-W to WFRs.

Furthermore, comparable to our findings, Martin-Prevel et al. [23] reported that a nonquantitative list-based 24-HR overreported FGDS—using 6 through 21 food subgroups—as compared with a quantitative 24-HR with ≥15 g/d minimum intake thresholds for food groups to count. The food groups most frequently overreported in rural Burkina Faso—using a 9-point FGDS—were “legumes and nuts,” “flesh foods,” “dark green leafy vegetables,” and “vitamin A-rich fruit and vegetables” [23]. Noteworthy for monitoring healthy diets globally, is that misreporting of food group consumption increased for more disaggregated food group-based metrics [23]—consequently hampering their descriptive utility for within and cross-country comparisons at the food subgroup-level.

In addition, analogous to our WFR data, quantitative 24-HRs in Ethiopia indicated that most consumers of the “pulses” food group ate ≥15 g/d [22]. Nevertheless, in the current study, we identified that specific food items, including *shiro* (powder) and mature beans, were consumed in nontrivial quantities (that is, ≥15 g/d) by only ∼50% of nonpregnant females, which led to substantial misclassification because of the criterion for foods to be counted toward MDD-W. Notably, *shiro* and (mature) beans, were also included as sentinel foods for the “pulses” food group on the DQQ. This may or may not be an important source of misreporting at the national level, depending on whether larger proportions of females consume *shiro* and beans in nontrivial quantities in other regions of Ethiopia.

Furthermore, Uyar et al. [22] reported that carrots—included in both the DQQ and our food list—were often consumed in amounts <15 g/d. These results are in contrast to the current study. To clarify, among consumers, the intake quantities of carrots were ≥15 g/d for >70% of females (data not shown) and carrots were one of only a few food items leading to underreporting of the “vitamin A-rich fruits and vegetables” food group (that is, false negatives).

Lastly, in parallel to a validation study in Nigeria and Vietnam [24], our WFR data indicates that fruits were almost exclusively consumed per item and thus in quantities ≥15 g/d, whereas rarely being used in mixed dishes (data not shown). Pastori et al. [24] subsequently concluded that fruit food groups are therefore less prone to recall biases (that is, more easily remembered by respondents). Our findings refute this claim in Ethiopia, as fruits—and other food items perceived as “healthy”—were major sources of food group intrusions during the enumeration of proxy data collection methods, in particular for list-based 24-HRs. Similarly, Rumpler et al. [25] reported that intrusions contributed to the greatest proportion of variance in error in the intake of meats (53%), which was closely followed by fruits and juices (46%).

Overreporting of food group consumption by proxy data collection methods is striking, as most previous studies assessing the relative validity of quantitative 24-HRs in low- and middle-income countries indicated underreporting of specific food groups, energy, and nutrient intakes [1]. To illustrate, among females in rural Ethiopia, Alemayehu et al. [26] showed that 36% of females omitted items, whereas 25% of quantitative 24-HRs contained foods that were not consumed according to WFRs, which were mainly drinks and irregularly consumed foods in both cases. Similarly, among mothers in rural Kenya, 31% omitted food items, especially fruits, whereas 19% of 24-HRs had intrusions (that is, food and drink items not consumed according to WFRs) [27]. Although the impacts of these omissions and intrusions on food group intake prevalence were not assessed in either study, the potential effects are likely to be larger in settings with low intrafood group diversity.

In the present study, the underlying reasons for the overreporting of fruit and vegetable food groups might be attributed to attitudes toward food groups perceived as “healthy,” underlying health and socio-economic status of females, and intrahousehold gender aspects of food distribution [28]. Moreover, in addition to respondent memory lapses and recalling intake beyond the 24-h period, instances of inappropriate manner of questioning during the 24 HRs by the enumerator, such as an excessively fast pace, leading questions, or judgmental comments, may lead to 2 other sources of respondent bias: social desirability (that is, the tendency to respond in such a way as to avoid criticism) and social approval (that is, the tendency to seek praise). To illustrate, there might have been an inclination of underreporting food groups perceived as “unhealthy” (for example, added sugars, sweets, and fats, as observed among females in rural Kenya [27]) and a selective overreporting of “healthy” food groups (for example, dark green leafy vegetables) [1]. Lastly, in particular for the list-based 24-HR, providing examples (that is, sentinel foods) might have acted as a prompt for reporting intake, but unintentionally led to intrusions.

Our study has some limitations that warrant caution. Single-day direct observations of dietary intake were undertaken solely as a means of validating same-day proxy data collection methods for MDD-W. Hence, subsequent food group consumption and MDD-W prevalence estimates do not reflect habitual intakes among the convenient sample of nonpregnant Ethiopian females, as single-day assessments ignore random within-person day-to-day and seasonal variability [29,30]. Furthermore, the presence of an enumerator during at-home and away-from-home food consumption might have led to individuals altering their eating habits [31]. However, the 8% MDD-W prevalence from WFR reported in the current study was similar to previous estimates from quantitative and nonquantitative 24-HRs among females in the same region [32,33]. Nonetheless, local dietary customs and diverging MDD-W estimates [that is, range of published means: 3%–82% [34,35] suggest that nonpregnant women in the Amhara region may not be representative of the general female population in Ethiopia. In addition, there are potential methodological issues inherent to the WFR that may contribute to differences in food group prevalence as compared with proxy data collection methods. For cooked mixed dishes, the conversion to amounts of individual ingredients relies on yield factors that are averages from the United States, which might have inflated the (small) number of errors related to the 15 g/d minimum quantity threshold for a food group to be counted as consumed. Moreover, because of the nature of the list-based 24-HR (that is, recall documented at the food group, not individual food item level), our analyses were unable to assess the extent to which specific food item misclassifications contributed to misreporting [11]. Lastly, there were minor systematic errors introduced by differential assignment of food codes (for example, Ethiopian kale coded as a “vitamin A-rich fruit and vegetable,” rather than as a “dark green leafy vegetable”) by WFR and list-based and open 24-HRs, but sensitivity analyses confirmed they did not affect our main conclusions (data not shown).

The current study design also has several strengths. Objective quantitative dietary assessments of all foods and beverages consumed from the first to last meal of the day, achieved by “shadowing” respondents at-home and out-of-home settings, removed (systematic) errors because of respondent biases and hence allowed the rigorous partitioning of food group and food item misclassifications linked to proxy data collection methods, which is not possible when using quantitative 24-HR as a reference method [36].

In conclusion, the observed overreporting of food group consumption by proxy data collection methods was predominantly due to respondent biases or misclassification because of the criterion for foods to be counted toward MDD-W (that is, food items generally consumed in nontrivial quantities in the sample, which are consumed by a limited number of respondents in <15 g/d), rather than the suboptimal development of the food list in Ethiopia. Therefore, to obtain precise, accurate, and subsequently reliable MDD-W estimates at (sub)national level, context-specific food lists development with local nutrition experts (that is, excluding food items frequently eaten in trivial quantities), questionnaire pilot testing, and also rigorous enumerator training is recommended, as per FAO’s updated MDD-W guidance.

## Author contributions

The authors’ responsibilities were as follows – GH-C: designed the research; GH-C: analyzed data and performed statistical analysis; GH-C, JPP: curated and managed data; GH-C, SH: wrote the article; GH-C, SH: have primary responsibility for final content; and all authors: read and approved the final manuscript.

## Conflict of interest

The authors report no conflicts of interest.

## Funding

Data collection was supported by the German Federal Ministry for Food and Agriculture (BMEL) under technical leadership from the Food and Agriculture Organization of the United Nations (FAO) and Ghent University (UGent). This study was supported the German Development Cooperation Agency (GIZ) – Knowledge for Nutrition (K4N). The funder had no role in the study design, data analysis, decision to publish, or preparation of the manuscript.

## Data availability

Anonymized data described in the manuscript, code book, and analytic code will be made available upon request pending review by the corresponding author and the signing of a data-sharing agreement with FAO.

## Disclaimer

The views expressed in this publication are those of the authors and do not necessarily reflect the views or policies of the FAO of the United Nations.
