# Supplementary material for: Minimum Dietary Diversity for Women: Partitioning Misclassifications by Proxy Data Collection Methods using Weighed Food Records as the Reference in Ethiopia
Source: Curr Dev Nutr. 2024 Jun 13;8(7):103792. doi: 10.1016/j.cdnut.2024.103792 (PMC11266992; doi:10.1016/j.cdnut.2024.103792)
Supplement: Multimedia component 1 [file mmc1.docx]

**Supplemental table 1. Overreported foods by list-based or open methods, as compared to weighed food record in Ethiopia (*n*=431)^1^**

|  | **Weighed food record** | | |  |  |
| --- | --- | --- | --- | --- | --- |
|  | **Consumers**  ***n* (%)** | **1-14 g/day**  ***n* (%)** | **≥15 g/day**  ***n* (%)** | **On food list** | ***Post-hoc***  **recommendation** |
|  |  | **Beans, peas, and lentils** | |  |  |
| *Shiro* (powder) | 229 (53.1) | 94 (41.0) | 135 (59.1) | Yes | Remove^2^ |
| Mature beans | 128 (29.7) | 59 (46.1) | 69 (54.0) | Yes | Remove^2^ |
| Peas | 68 (15.8) | 11 (16.2) | 57 (83.8) | Yes | Keep |
| Lentils | 143 (33.2) | 23 (16.1) | 120 (83.9) | Yes | Keep |
|  |  | **Other vegetables** | |  |  |
| Onion | 426 (98.8) | 39 (9.15) | 387 (90.8) | No | Add |
| Green pepper | 144 (33.4) | 134 (93.1) | 10 (6.94) | Yes | Remove^2^ |
| Tomato | 235 (54.5) | 24 (10.2) | 211 (89.8) | Yes | Keep |

^1^All underreported foods were included on the food list, except lemon, which was consumed by 3.2% of non-pregnant women, respectively. To determine whether specific food items should have been included or excluded on the predefined Ethiopian food list, we set the arbitrary but stringent criteria that, among consumers of the food item in question, at least 70% had intakes ≥15 g/day.

^2^To reduce type I errors (i.e., false positive values). To err on the side of caution, specificity (i.e., true negative rate) was weighted higher than sensitivity (i.e., true positive rate) during Minimum Dietary Diversity for Women validation.
